# Associations of dietary inflammatory index with incident cardiovascular outcomes and all-cause mortality in patients with prediabetes and type 2 diabetes mellitus: A prospective study of data from the UK Biobank

**DOI:** 10.1371/journal.pone.0360954

**Published:** 2026-11-11

**Authors:** Ya Shi, Jingjing Zeng, Menglin Fan, Bo Chen, Zhaoxia Zhang, Bo Yang, Yang Tian, Guigao Guo, Nan Wang, Ying Xing, Shaoyong Xu

**Affiliations:** 1 Department of Endocrinology, Xi’an Daxing Hospital Affiliated to Yan’an University, Xi’an, Shaanxi, China; 2 Center for Clinical Evidence-Based and Translational Medicine, Xiangyang Central Hospital, Affiliated Hospital of Hubei University of Arts and Science, Xiangyang, Hubei, China; 3 Department of Cardiology, Xijing Hospital, The Fourth Military Medical University, Xi’an, Shaanxi, China; 4 Department of Neurosurgery, Tangdu Hospital, Fourth Military Medical University, Xi’an, Shaanxi, China; 5 Department of Endocrinology, Xiangyang Central Hospital, Affiliated Hospital of Hubei University of Arts and Science, Xiangyang, Hubei, China; University of Montenegro-Faculty of Medicine, MONTENEGRO

## Abstract

**Background:**

While the dietary inflammatory index (DII) is linked to cardiovascular disease (CVD) and mortality in the general population, its role in individuals with prediabetes and type 2 diabetes (T2D) remains understudied. Given the elevated inflammatory burden in this high-risk group, we aimed to examine the association between energy-adjusted DII (E-DII) and incident cardiovascular outcomes and all-cause mortality using a prospective cohort from the UK Biobank.

**Methods:**

We conducted a prospective cohort study using UK Biobank data, including 18,193 individuals with prediabetes and 6,469 with T2D, all free of cardiovascular disease and cancer at baseline. The E-DII was derived from 24-hour dietary recall data, incident CVD and all-cause mortality were ascertained through linked hospital and death records. Cox proportional hazards models estimated hazard ratios (HRs) and 95% confidence intervals (CIs), with a median duration of 12.2 years (IQR: 11.5–13.0).

**Results:**

Among participants with T2D, an energy-adjusted DII > 1 (vs. ≥−1 to ≤1) was associated with a higher risk of cardiovascular events (HR 1.24, 95% CI 1.00–1.53) and all-cause mortality (HR 1.36, 95% CI 1.01–1.84). The association with cardiovascular mortality was not statistically significant (HR 1.50, 95% CI 0.76–2.94). No significant associations were observed among participants with prediabetes.

**Conclusions:**

A more pro-inflammatory dietary pattern, as measured by the E-DII, is independently associated with increased risks of incident cardiovascular events and all-cause mortality in individuals with T2D. These findings support the integration of DII into nutritional risk assessment for diabetic populations and highlight the potential of dietary modification to improve long-term outcomes.

## Introduction

The global prevalence of T2D and prediabetes is increasing rapidly. According to the International Diabetes Federation (IDF), diabetes affected 11.11% of the global adult population (589 million individuals) in 2024 and is projected to rise to 12.96% (853 million) by 2050 [1]. T2D accounts for more than 90% of diabetes cases worldwide [2]. Prediabetes represents an equally substantial public health burden, with an estimated 548.7 million adults aged 20–79 years living with prediabetes globally in 2024, representing approximately 10.7% of the adult population [1]. The annual progression rate from prediabetes to T2D is approximately 5.7%, with higher rates observed among individuals with obesity or a family history of diabetes [1,3]. Prediabetes is associated with increased risks of developing T2D, cardiovascular events, and all-cause mortality. First-line therapy for prediabetes is lifestyle modification (including weight loss and increased physical activity) and, for some individuals, metformin. Lifestyle modification generally confers greater benefits than metformin [4,5]. The cardiovascular and mortality burden associated with T2D is substantial. Individuals with T2D face more than a twofold increased risk of cardiovascular disease (CVD) compared to those without diabetes [6]. Moreover, T2D is associated with significantly elevated all-cause mortality, with a hazard ratio of approximately 1.95 relative to non-diabetic populations [7]. This excess risk extends to the prediabetic state, which is independently associated with higher risks of CVD and all-cause mortality [8–10]. Although lifestyle interventions represent the cornerstone of management, their long-term impact on cardiovascular outcomes and all-cause mortality in prediabetic individuals remains uncertain [11]. These findings underscore the importance of early identification and risk-stratified management of both T2D and prediabetes to mitigate cardiovascular morbidity and mortality.

Diet is a key modifiable factor in diabetes management and may substantially influence CVD risk in this population [12]. A high-quality dietary pattern can improve glycaemic control and reduce cardiometabolic risk factors, thereby lowering the risk of cardiovascular complications [13]. While multiple dietary patterns have been associated with CVD risk among individuals with T2D and prediabetes [13–15], the underlying mechanisms are not fully understood. Chronic low-grade systemic inflammation has emerged as a critical pathophysiological link connecting dietary intake, glucose dysregulation, and cardiovascular complications in these populations. However, traditional dietary assessment methods primarily focus on macronutrient composition or adherence to specific dietary patterns (e.g., Mediterranean, plant-based diets) and do not fully capture the overall pro-inflammatory potential of an individual’s diet.

To address this limitation, the Dietary Inflammatory Index (DII), developed by Shivappa et al., was designed to quantify the inflammatory potential of the overall diet based on empirical evidence regarding associations between dietary components and inflammatory biomarkers [16–19]. Higher DII scores (i.e., more pro-inflammatory diets) have been associated with diabetes prevalence and with higher risks of all-cause and cardiovascular mortality in populations with diabetes [20–22]. By contrast, evidence regarding the DII-mortality association in prediabetic individuals is emerging but remains limited [23].

Compared with the original DII, the E-DII accounts for between-person differences in total energy intake and may provide a more comparable measure across individuals [24,25]. Therefore, using UK Biobank data, we investigated the associations between E-DII and incident cardiovascular outcomes and all-cause mortality among participants with prediabetes and T2D.

## Methods

### Design and participants

The UK Biobank is a well-designed, large-scale prospective cohort study that successfully recruited over 500,000 participants aged between 40 and 69 years from 2006 to 2010. The study has so far collected a large amount of phenotypic and genotypic information from the participants and will continue to collect relevant data. These data are collected by means of questionnaires, physical measurements, biological samples, whole-genome sequencing, and longitudinal follow-up records, and the collected data inform of a wide variety of health outcomes [26,27]. To date, more than 200,000 participants have filled in detailed dietary web questionnaires up to four times, and around 20,000 participants have completed repeated data collection of the baseline assessment [28,29]. More information about the UK Biobank protocol can be accessed on the website (http://www.ukbiobank.ac.uk).This study is a retrospective analysis using de-identified data from the UK Biobank resource. Data were accessed for research purposes on 15th March 2024. The UK Biobank study was approved by the North West Multi-centre Research Ethics Committee (REC reference: 21/NW/0157), and all procedures were conducted in accordance with the 1964 Declaration of Helsinki and its subsequent amendments. All participants provided written informed consent prior to enrolment, and no personally identifiable information is included in this manuscript.

### Definitions of prediabetes and type 2 diabetes mellitus

Participants with prediabetes were defined by the presence of impaired fasting glucose and/or a hemoglobin A1c (HbA1c) of 5.7%–6.4% (39–47 mmol/mol) according to the 2021 diagnostic criteria of the American Diabetes Association. Participants with type 2 diabetes mellitus included those who had been diagnosed by a physician during the touchscreen questionnaire, those taking insulin and hypoglycemic medication, or those with HbA1c ≥ 6.5% (48 mmol/mol) at baseline [30].

### Diet inflammatory index

The intake of energy and nutrients was calculated using the 5th edition of “The Composition of Foods” by McCance and Widdowson [31]. According to the scoring algorithm proposed by Shivappa et al., the present study considered 18 foods and nutrients included in the UK Biobank when calculating the energy-adjusted DII, including alcohol, carbohydrates, cellulose, folic acid, saturated fat, polyunsaturated fat, protein, total fat, vitamin B12, vitamin B6, iron, magnesium, vitamin C, vitamin E, tea, garlic, onion, and total energy. By summing the DII scores of these 18 foods and nutrients, the total DII score was obtained. Inflammation is closely related to the total energy consumption from the diet, and many studies have shown a strong negative correlation between the DII and total energy intake. Therefore, the energy-adjusted DII was developed. The energy-adjusted DII is calculated according to the following steps. First, the reported intake was converted into a relative value; that is, the intake per 1,000 kcal/day. Second, in line with the global comparison database, the energy adjustment value per 1,000 kcal/day was adopted. All analyses were based on the energy-adjusted DII value. The energy-adjusted DII, as a continuous variable, is divided into three categories: very/moderately anti-inflammatory (<−1), neutral (≥−1 to ≤1), and very/moderately pro-inflammatory (>1). This classification method has been applied in previous studies [28].

### Study population

The baseline population comprised 502,392 subjects, including 411,266 without diabetes mellitus, 4,777 with type 1 diabetes mellitus, and 86,349 with type 2 diabetes mellitus or prediabetes. At baseline, 3587 individuals reported having cardiovascular disease and 2,391 reported having cancer. Overall, 535 individuals were excluded owing to insufficient dietary records. Therefore, a total of 24,662 participants were included in the final analysis, including 6,469 patients with type 2 diabetes mellitus and 18,193 with prediabetes (Fig 1).

**Fig 1:**
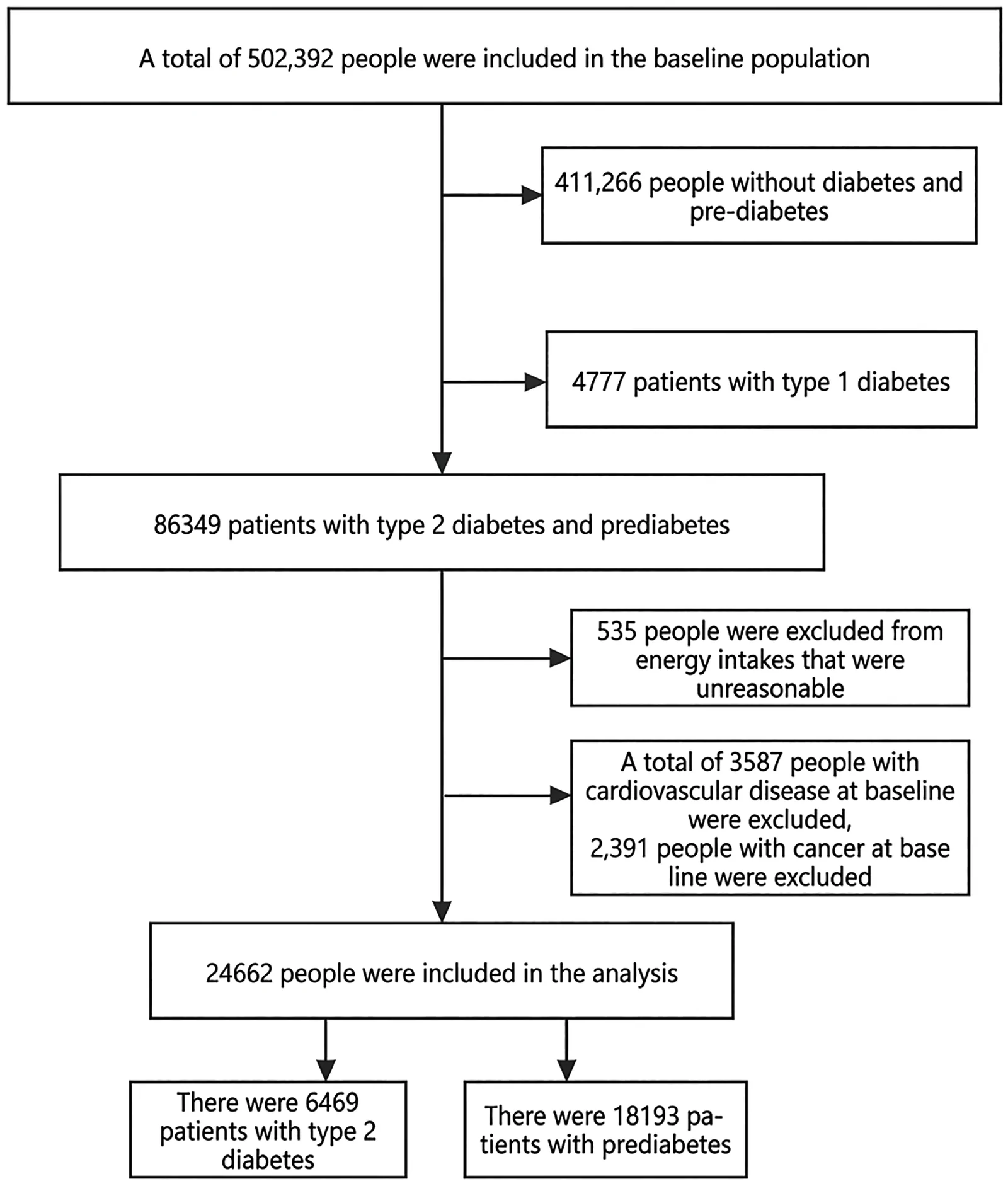
Flowchart of participant selection.

### Cardiovascular outcomes and all-cause mortality

Cardiovascular outcomes encompassed a series of cardiovascular events, including the first reported cases of cardiovascular disease and mortality caused by cardiovascular disease. Incident events and mortality outcomes were defined using the following International Classification of Diseases-10 codes: I20, I21, I22, I23, I24, I25, I46, I50, I60, I61, I162, I63, I64, and mortality due to cardiovascular disease without specified bleeding or infarction. Data on these outcomes were obtained from hospital and death databases. The date and cause of death were obtained from the death certificates held by the National Health Service (NHS) Information Center (covering England and Wales) and the National Health Service Center Scottish Registry (covering Scotland). The date and cause of hospitalization were determined through Health Statistics records (covering England and Wales) and the Scottish Morbidity Record (SMR01) (covering Scotland). The time point when the patient first experienced any of the above events during the follow-up period was recorded as the date of the event occurrence. For each study subject, the length of follow-up to the endpoint was calculated based on the date of the occurrence of the endpoint event, loss to follow-up, or death, whichever came first.

### Covariates

The baseline age of the participants was determined by their date of birth and the assessment at baseline. The socioeconomic status was determined based on the postal code of the participant’s place of residence and was assessed using the Townsend score. Ethnic information was collected through self-reporting and classified as White, Black, Asian, or other. Smoking status was also determined through self-reporting and divided into never smoker, former smoker, and current smoker. Height, weight, blood pressure, glycated hemoglobin (HbA1c), blood lipids, and creatinine concentration were all measured following standardized procedures. Body mass index (BMI) was calculated by dividing the weight in kilograms by height in meters squared. The diagnostic criteria for hypertension included previous diagnosis by a doctor, treatment with antihypertensive drugs, or an average blood pressure ≥140/90 mmHg measured on three different dates. More detailed information about the measurement methods can be found on the official website of the UK Biobank (http://www.ukbiobank.ac).

### Statistical analysis

The participants were divided into three groups according to their energy-adjusted DII values, as follows: <−1 (very/moderately anti-inflammatory), ≥−1 to ≤1 (neutral), and >1 (very/moderately pro-inflammatory). Continuous data are presented as the mean ± standard deviation depending on the normality of their distribution, while categorical data are presented as number (%). The chi-square test or analysis of variance was used to compare the demographic and clinical characteristics at baseline among the different energy-adjusted DII groups. To assess the association between the energy-adjusted DII and the risk of major endpoint events, we constructed Cox proportional-hazards regression models to calculate the hazard ratios (HRs) and 95% confidence intervals (CIs). When adjusting for potential confounding factors, we used three different models based on the guidance of the previous literature. Model 0 was unadjusted; Model 1 was adjusted for sociodemographic factors (age, sex, Townsend deprivation index, and race); and Model 2 was adjusted for the same covariates as Model 1, as well as BMI, physical activity, smoking status, alcohol intake frequency, sleep chronotype, and baseline HbA1c. For patients with diabetes mellitus, we adjusted for the variables in Model 2, as well as insulin treatment and baseline diabetes mellitus duration. These covariates were selected based on their known associations with the study exposure and outcome [32]. To test the robustness of the results, we also conducted a series of subgroup and sensitivity analyses. Restricted cubic splines were used to explore the non-linear relationship between the energy-adjusted DII and outcomes.

## Results

### Baseline characteristics of the participants

A total of 24,662 participants with complete data were included (Fig 1), with a mean age of 58.71 ± 6.97 years, and 48.00% of the participants were male. The vast majority of the participants were White (87.17%), while Asian/Asian British and Black/Black British accounted for 4.03% and 2.84% of the participants, respectively. Based on the Townsend index, the mean socioeconomic status score of the participants was −1.32 ± 2.30, indicating that most participants were from a relatively favorable economic background. The mean HbA1c was 6.14% ± 0.79%, suggesting that the overall blood glucose control of the participants was good (Table 1).

**Table 1:** Demographic and baseline characteristics of the participants according to the energy-adjusted DII among patients with prediabetes or type 2 diabetes mellitus.

Characteristics	Total (N = 24,662)	Anti-inflammatory (N = 9,465)	Neutral (N = 12,436)	Pro-inflammatory (N = 2,761)	P
**Prediabetes**	18,193	6,923	9,192	2,078	
Age, years	58.80 ± 6.87	59.70 ± 6.27	58.59 ± 6.98	56.71 ± 7.71	< 0.001
Gender, male (%)	7,924 (43.56)	2,452 (35.42)	4413 (48.01)	1059 (50.96)	< 0.001
Ethnicity					< 0.001
White	15,982 (87.85)	6,124 (88.46)	8126 (88.40)	1732 (83.35)	
Asian or Asian British	641 (3.52)	246 (3.55)	320 (3.48)	75 (3.61)	
Black or Black British	488 (2.68)	153 (2.21)	231 (2.51)	104 (5.00)	
Other	1,082 (5.95)	400 (5.78)	515 (5.60)	167 (8.04)	
Townsend deprivation index	−1.42 ± 2.95	−1.60 ± 2.84	−1.45 ± 2.95	−0.71 ± 3.17	< 0.001
BMI, kg/m^2^	28.50 ± 5.25	27.99 ± 5.13	28.61 ± 5.17	29.72 ± 5.75	< 0.001
BMI categories					< 0.001
< 18.5 kg/m^2^	96 (0.53)	39 (0.56)	44 (0.48)	13 (0.63)	
18.5–24.9 kg/m^2^	4,652 (25.57)	2,031 (29.34)	2243 (24.40)	378 (18.19)	
25–29.9 kg/m^2^	7,469 (41.05)	2,826 (40.82)	3829 (41.66)	814 (39.17)	
≥ 30 kg/m^2^	5,976 (32.85)	2,027 (29.28)	3076 (33.46)	873 (42.01)	
Physical activity, MET-h/wk	41.37 ± 42.48	44.03 ± 42.61	39.98 ± 42.02	38.54 ± 43.61	< 0.001
Smoking status					< 0.001
Never	9583 (52.67)	3876 (55.99)	4745 (51.62)	962 (46.29)	
Previous	6481 (35.62)	2532 (36.57)	3287 (35.76)	662 (31.86)	
Current	2129 (11.70)	515 (7.44)	1160 (12.62)	454 (21.85)	
Alcohol consumption					< 0.001
Never	821 (4.51)	313 (4.52)	398 (4.33)	110 (5.29)	
Previous	685 (3.77)	242 (3.50)	327 (3.56)	116 (5.58)	
Occasionally	4597 (25.27)	1658 (23.95)	2251 (24.49)	688 (33.11)	
Weekly	8361 (45.96)	3351 (48.40)	4186 (45.54)	824 (39.65)	
Daily	3701 (20.34)	1354 (19.56)	2012 (21.89)	335 (16.12)	
Unclear	28 (0.15)	5 (0.07)	18 (0.20)	5 (0.24)	
Sleep chronotype					< 0.001
Morning	10337 (56.82)	4197 (60.62)	5075 (55.21)	1065 (51.25)	
Evening	6113 (33.60)	2130 (30.77)	3163 (34.41)	820 (39.46)	
Unclear	1743 (9.58)	596 (8.61)	954 (10.38)	193 (9.29)	
HbA1c, %	5.90 ± 0.17	5.90 ± 0.17	5.90 ± 0.17	5.91 ± 0.17	0.016
**Diabetes**	6,469	2,542	3,244	683	
Age, years	58.47 ± 7.24	59.36 ± 6.88	58.22 ± 7.25	56.30 ± 7.90	< 0.001
Gender, male (%)	3914 (60.50)	1420 (55.86)	2061 (63.53)	433 (63.40)	< 0.001
Ethnicity					0.033
White	5517 (85.28)	2142 (84.26)	2804 (86.44)	571 (83.60)	
Asian or Asian British	354 (5.47)	160 (6.29)	163 (5.02)	31 (4.54)	
Black or Black British	213 (3.29)	88 (3.46)	98 (3.02)	27 (3.95)	
Other	385 (5.95)	152 (5.98)	179 (5.52)	54 (7.91)	
Townsend deprivation index	−1.03 ± 3.12	−1.21 ± 3.07	−1.01 ± 3.08	−0.40 ± 3.35	< 0.001
BMI, kg/m^2^	31.20 ± 5.89	30.55 ± 5.76	31.32 ± 5.80	33.02 ± 6.38	< 0.001
BMI categories					< 0.001
< 18.5 kg/m^2^	5 (0.08)	2 (0.08)	3 (0.09)	0 (0)	
18.5–24.9 kg/m^2^	766 (11.84)	371 (14.59)	344 (10.60)	51 (7.47)	
25–29.9 kg/m^2^	2350 (36.33)	975 (38.36)	1180 (36.37)	195 (28.55)	
≥ 30 kg/m^2^	3348 (51.75)	1194 (46.97)	1717 (52.93)	437 (63.98)	
Physical activity, MET-h/wk	35.83 ± 39.55	39.46 ± 41.27	33.89 ± 37.67	31.55 ± 40.71	< 0.001
Smoking status					< 0.001
Never	3118 (48.20)	1274 (50.12)	1511 (46.58)	333 (48.76)	
Previous	2766 (42.76)	1080 (42.49)	1430 (44.08)	256 (37.48)	
Current	585 (9.04)	188 (7.40)	303 (9.34)	94 (13.76)	
Alcohol consumption					< 0.001
Never	372 (5.75)	159 (6.25)	163 (5.02)	50 (7.32)	
Previous	343 (5.30)	138 (5.43)	165 (5.09)	40 (5.86)	
Occasionally	1972 (30.48)	768 (30.21)	946 (29.16)	258 (37.77)	
Weekly	2682 (41.46)	1072 (42.17)	1378 (42.48)	232 (33.97)	
Daily	1094 (16.91)	403 (15.85)	588 (18.13)	103 (15.08)	
Unclear	6 (0.09)	2 (0.08)	4 (0.12)	0 (0)	
Sleep chronotype					< 0.001
Morning	3571 (55.20)	1455 (57.24)	1777 (54.78)	339 (49.63)	
Evening	2269 (35.07)	824 (32.42)	1167 (35.97)	278 (40.70)	
Unclear	629 (9.72)	263 (10.35)	300 (9.25)	66 (9.66)	
HbA1c, %	6.88 ± 1.30	6.79 ± 1.24	6.93 ± 1.32	6.98 ± 1.37	< 0.001
Diabetes duration, year	4.39 ± 6.27	4.55 ± 6.62	4.33 ± 6.11	4.04 ± 5.60	0.127
Insulin treatment, yes (%)	498 (7.70)	189 (7.44)	252 (7.77)	57 (8.35)	0.714

Values are presented as the mean ± standard deviation or number (%). DII, Dietary Inflammation Index; BMI, body mass index; MET, metabolic equivalent of physical activity; HbA1c, glycated hemoglobin.

In the analysis by energy-adjusted DII group, there were significant differences in demographic characteristics and lifestyle factors among the groups. Table 1 shows that compared with the participants in the neutral or very/moderately anti-inflammatory groups, those in the very/moderately pro-inflammatory group were younger, more likely to be male, had a higher Townsend deprivation index, had a higher BMI, performed less physical activity, and were more likely to never drink alcohol. Furthermore, the participants with type 2 diabetes mellitus in the very/moderately anti-inflammatory group had a higher HbA1c level (Table 1).

Table 2 shows the associations between cardiovascular outcome events, cardiovascular mortality, and all-cause mortality in patients with prediabetes and type 2 diabetes mellitus. We conducted follow-up of cardiovascular disease events for a median of 12.07 (11.45–12.80) years and cardiovascular mortality for a median of 12.27 (11.72–13.03) years.

**Table 2:** Associations of the energy-adjusted DII with the risk of cardiovascular disease, cardiovascular mortality, and all-cause mortality in patients with prediabetes or type 2 diabetes mellitus.

	Cases of events/N (%)	Person-years	HR (95% CI)		
			Crude model	Model 1	Model 2
**Prediabetes**					
**Cardiovascular events**	2153/18193 (11.83)	211,031			
Anti-inflammatory	811/6923 (11.71)	80,541	0.98 (0.90–1.08)	1.02 (0.93–1.12)	1.05 (0.94–1.16)
Neutral	1089/9192 (11.85)	106,581	Ref	Ref	Ref
Pro-inflammatory	253/2078 (12.18)	23,907	1.04 (0.91–1.19)	1.12 (0.97–1.28)	0.97 (0.83–1.14)
**Cardiovascular mortality**	122/18193 (0.67)	223,072			
Anti-inflammatory	39/6923 (0.56)	85,087	0.78 (0.53–1.16)	0.79 (0.53–1.18)	0.96 (0.60–1.52)
Neutral	66/9192 (0.72)	112,665	Ref	Ref	Ref
Pro-inflammatory	17/2078 (0.82)	25,319	1.15 (0.68–1.97)	1.23 (0.72–2.11)	0.97 (0.50–1.87)
**All-cause mortality**	1071/18193 (5.89)	223,072			
Anti-inflammatory	383/6923 (5.53)	85,087	0.94 (0.82–1.07)	0.94 (0.82–1.07)	1.00 (0.86–1.16)
Neutral	540/9192 (5.87)	112,665	Ref	Ref	Ref
Pro-inflammatory	148/2078 (7.12)	25,319	1.23 (1.02–1.47)^*^	1.33 (1.11–1.59)^**^	1.20 (0.98–1.48)
**Diabetes**					
**Cardiovascular events**	1154/6469 (17.84)	72,221			
Anti-inflammatory	452/2542 (17.78)	25,454	1.03 (0.91–1.16)	1.00 (0.88–1.13)	1.02 (0.88–1.17)
Neutral	562/3244 (17.32)	36,291	Ref	Ref	Ref
Pro-inflammatory	140/683 (20.50)	7,475	1.21 (1.01–1.46)^*^	1.30 (1.08–1.57)^**^	1.24 (1.00–1.53)^*^
**Cardiovascular mortality**	89/6469 (1.38)	78,224			
Anti-inflammatory	29/2542 (1.14)	30,774	0.77 (0.49–1.23)	0.75 (0.47–1.20)	0.78 (0.47–1.31)
Neutral	48/3244 (1.48)	39,267	Ref	Ref	Ref
Pro-inflammatory	12/683 (1.76)	8,181	1.21 (0.64–2.27)	1.33 (0.70–2.51)	1.50 (0.76–2.94)
**All-cause mortality**	559/6469 (8.64)	78,224			
Anti-inflammatory	217/2542 (8.54)	30,774	1.01 (0.85–1.21)	0.99 (0.83–1.18)	1.09 (0.88–1.33)
Neutral	275/3244 (8.48)	39,267	Ref	Ref	Ref
Pro-inflammatory	67/683 (9.81)	8,181	1.18 (0.90–1.54)	1.28 (0.98–1.68)	1.36 (1.01–1.84)^*^

Multivariate Cox proportional-hazards regression Model 1 was adjusted for age (continuous), sex, ethnicity, and Townsend deprivation index. Model 2 was adjusted for the variables adjusted in Model 1 plus BMI (categorical), physical activity, smoking status, alcohol intake frequency, sleep chronotype, and baseline HbA1c. For participants with type 2 diabetes mellitus, the model was adjusted for the variables adjusted in Model 2 plus insulin treatment and diabetes mellitus duration at baseline. BMI, body mass index; HR, hazard ratio; CI, confidence interval; DII, Dietary Inflammatory Index. ^*^P < 0.05; ^**^P < 0.01.

### Association of the energy-adjusted DII with cardiovascular outcomes and all-cause mortality

When analyzing patients with prediabetes, we observed that the association between the very/moderately pro-inflammatory diet pattern and all-cause mortality risk varied among the different models. In the adjusted model (Model 2), the risk of all-cause mortality in the very/moderately pro-inflammatory group was 1.20 (95% CI 0.98–1.48). Moreover, compared with the control group, the very/moderately pro-inflammatory diet pattern was not significantly correlated with the risk of cardiovascular events and cardiovascular mortality (Table 2). The results of the subgroup analysis showed that the associations of the energy-adjusted DII with the risk of cardiovascular disease, cardiovascular mortality, and all-cause mortality in patients with prediabetes were inconsistent in the different subgroups, and most of the associations became non-significant after multivariate adjustment (S1 Table). The non-linear associations of the energy-adjusted DII with all-cause mortality and cardiovascular disease were not significant (Fig 2).

**Fig 2:**
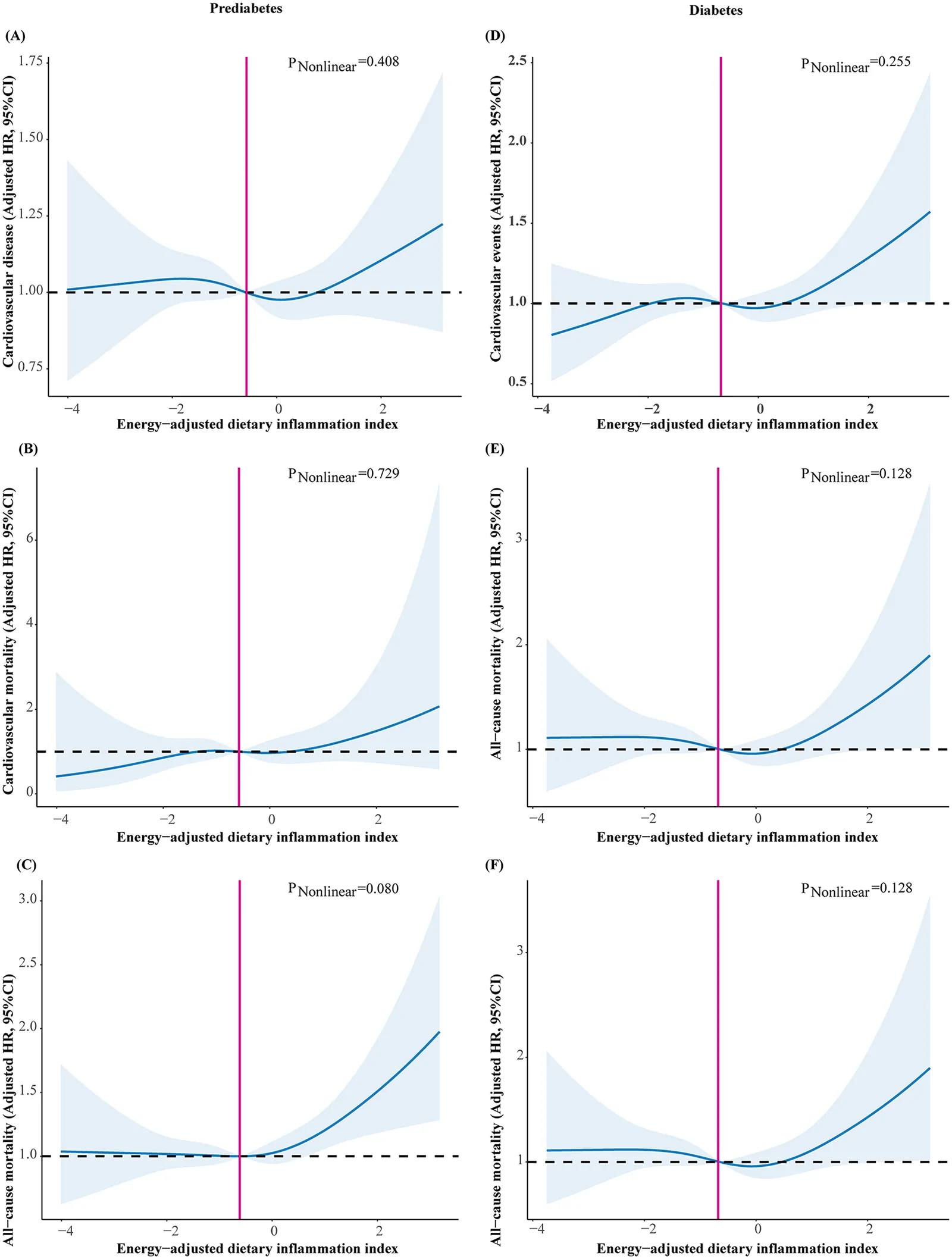
Restricted cubic splines of the associations between the energy-adjusted DII and the risk of cardiovascular disease, cardiovascular mortality, and all-cause mortality in patients with prediabetes or type 2 diabetes mellitus. DII, Dietary Inflammatory Index.

In the group of patients with type 2 diabetes mellitus, the results indicated that the very/moderately pro-inflammatory diet pattern was associated with an increased risk of cardiovascular disease events and all-cause mortality. Specifically, in Model 2, we found that the HR of cardiovascular disease events in the very/moderately pro-inflammatory group was 1.24 (95% CI 1.00–1.53, P < 0.05), and the HR of all-cause mortality was 1.36 (95% CI 1.01–1.84, P < 0.05) (Table 2). However, there was no significant association between the very/moderately pro-inflammatory group and cardiovascular mortality. In addition, we conducted an in-depth analysis of different subgroups and found that in patients aged <60 years, the very/moderately pro-inflammatory diet pattern was associated with an increased risk of cardiovascular disease events, with an HR of 1.45 (95% CI 1.05–1.99). In the subgroups aged ≥60 years, of the male sex, with a BMI ≥ 30 kg/m2, and with a diabetes mellitus duration ≥5 years, the very/moderately pro-inflammatory group was associated with an increased risk of all-cause mortality. The association of each covariate included in the subgroup analysis is shown in S2 Table. The non-linear associations of the energy-adjusted DII with all-cause mortality and cardiovascular disease were not significant (Fig 2).

To test the robustness of the research results, we conducted a sensitivity analysis. In the unadjusted multivariate Cox proportional-hazards regression model, a positive association was observed between the very/moderately pro-inflammatory diet pattern and an increased risk of all-cause mortality in patients with prediabetes. After adjusting for age, sex, ethnicity, Townsend deprivation index, BMI, physical activity, smoking status, alcohol intake frequency, sleep chronotype, and baseline HbA1c (Model 2), the very/moderately pro-inflammatory diet pattern was still significantly associated with the risk of all-cause mortality (1.34, 95% CI 1.10–1.63). In patients with type 2 diabetes mellitus, the very/moderately pro-inflammatory diet pattern was associated with an increased risk of cardiovascular disease events and all-cause mortality. In Model 2, the HR of cardiovascular disease events was 1.22 (95% CI 1.01–1.49), and the HR of all-cause mortality was 1.34 (95% CI 1.01–1.77) (Table 3).

**Table 3:** Associations of the energy-adjusted DII with the risk of cardiovascular disease, cardiovascular mortality, and all-cause mortality in patients with prediabetes or type 2 diabetes mellitus.

	HR (95% CI)		
	Crude model	Model 1	Model 2
**Prediabetes**			
**Cardiovascular events**			
Anti-inflammatory	1.06 (0.97−1.16)	1.00 (0.91−1.09)	1.04 (0.94−1.15)
Neutral	Ref	Ref	Ref
Pro-inflammatory	0.97 (0.85−1.11)	1.10 (0.96−1.26)	1.01 (0.87−1.18)
**Cardiovascular mortality**			
Anti-inflammatory	0.92 (0.61−1.38)	0.83 (0.55−1.25)	0.76 (0.47−1.23)
Neutral	Ref	Ref	Ref
Pro-inflammatory	1.63 (1.01−2.61)	1.91 (1.19−3.08)	1.25 (0.69−2.28)
**All-cause mortality**			
Anti-inflammatory	0.96 (0.84−1.09)	0.90 (0.79−1.03)	0.95 (0.82−1.10)
Neutral	Ref	Ref	Ref
Pro-inflammatory	1.27 (1.07−1.51)	1.45 (1.22−1.73)	1.34 (1.10−1.63)
**Diabetes**			
**Cardiovascular events**			
Anti-inflammatory	0.97 (0.85−1.10)	0.94 (0.83−1.07)	0.95 (0.82−1.11)
Neutral	Ref	Ref	Ref
Pro-inflammatory	1.16 (0.98−1.37)	1.28 (1.08−1.52)	1.22 (1.01−1.49)
**Cardiovascular mortality**			
Anti-inflammatory	1.16 (0.73−1.83)	1.10 (0.69−1.74)	1.15 (0.70−1.91)
Neutral	Ref	Ref	Ref
Pro-inflammatory	1.43 (0.79−2.57)	1.63 (0.90−2.94)	1.53 (0.79−2.98)
**All-cause mortality**			
Anti-inflammatory	1.02 (0.85−1.23)	0.97 (0.81−1.17)	1.11 (0.90−1.37)
Neutral	Ref	Ref	Ref
Pro-inflammatory	1.15 (0.90−1.47)	1.31 (1.02−1.68)	1.34 (1.01−1.77)

Multivariate Cox proportional-hazards regression Model 1 was adjusted for age (continuous), sex, ethnicity, and Townsend deprivation index. Model 2 was adjusted for the variables adjusted in Model 1 plus BMI (categorical), physical activity, smoking status, alcohol intake frequency, sleep chronotype, and baseline HbA1c. For participants with diabetes mellitus, the model was adjusted for the variables adjusted in Model 2 plus insulin treatment and diabetes duration at baseline. BMI, body mass index; HbA1c, glycated hemoglobin; HR, hazard ratio; CI, confidence interval.

## Discussion

In this prospective cohort study of UK Biobank participants with a median follow-up of 12.2 years, we examined 6,469 participants with established T2D and 18,193 with prediabetes. A higher E-DII was significantly associated with increased risks of incident cardiovascular events and all-cause mortality among those with T2D. By contrast, no significant associations were observed between E-DII and these outcomes in the prediabetes group. These findings indicate that a more pro-inflammatory dietary pattern is independently linked to worse cardiovascular outcomes and elevated mortality risk specifically in individuals with established T2D.

Numerous studies have confirmed the association between the DII and cardiovascular risk as well as mortality in the general population [33–35]. However, evidence specifically addressing the DII-cardiovascular/mortality association in individuals with T2D and prediabetes remains limited. While previous investigations reported positive associations between pro-inflammatory diets and adverse outcomes in diabetic populations [20–22], our study notably extends this landscape in comparison to recent findings by Chen et al., who investigated the DII-mortality link specifically within diagnosed diabetes and focused on diabetes duration as an effect modifier. While their work provided valuable insights into post-diagnosis management, our research broadens the perspective to the “prediabetes-diabetes continuum” by incorporating a large prediabetic cohort. This allows us to assess how risk profiles shift as glucose tolerance deteriorates, filling a gap in early-stage prevention literature. Furthermore, unlike studies focusing primarily on mortality, our inclusion of non-fatal cardiovascular events (such as myocardial infarction and stroke) as hard endpoints provides a more comprehensive assessment of the DII’s impact on morbidity and long-term disability—factors that are often as clinically significant as mortality in chronic disease progression. Methodologically, the use of the energy-adjusted DII (E-DII) further strengthens these observations by reflecting dietary quality rather than caloric quantity. In our cohort, these associations remained significant in participants with T2D after adjustment for conventional risk factors.

Notably, the absence of significant associations in the prediabetes group contrasts with some prior studies. Although the NHANES III analysis by Deng et al. reported an association between the DII and mortality in prediabetic individuals [23], our UK Biobank cohort did not replicate this finding. This discordance may reflect important differences in study populations (e.g., age distribution, ethnicity, baseline health status, comorbidity patterns), dietary assessment methodologies, definitions of prediabetes, or follow-up duration. These findings are consistent with a threshold effect hypothesis, whereby the clinical impact of dietary inflammation on cardiovascular events and all-cause mortality may become apparent only in the context of more advanced glucose dysregulation, such as established T2D. While prediabetes is indeed associated with chronic low-grade systemic inflammation and elevated cardiovascular risk [36,37], the inflammatory burden at this earlier disease stage may be insufficient to amplify the deleterious effects of a pro-inflammatory diet on clinical outcomes.Alternatively, individuals with prediabetes may retain greater metabolic capacity to compensate for dietary inflammation or demonstrate better adherence to concurrent lifestyle modifications, both of which could attenuate the long-term impact of a pro-inflammatory diet on clinical outcomes. This observation underscores an important therapeutic window: early dietary intervention in the prediabetic stage, prior to the establishment of persistent low-grade inflammation and metabolic dysfunction, may represent a particularly effective prevention strategy.

Extensive pathophysiological evidence robustly supports a mechanistic link between pro-inflammatory dietary patterns and adverse outcomes in T2D. T2D is intimately associated with chronic low-grade systemic inflammation, which represents one of its cardinal pathophysiological features [38,39]. Chronic inflammatory processes not only drive the initiation and progression of T2D but also contribute substantially to the development of its major complications [40,41]. These findings support the hypothesis that the E-DII may provide clinically meaningful prognostic information for cardiovascular risk stratification in individuals with T2D. Specifically, among T2D patients, E-DII scores above the median were independently associated with a 24% increased risk of incident cardiovascular events and a 36% increased risk of all-cause mortality. Accordingly, dietary interventions specifically targeting a reduction in dietary inflammatory load—through increased consumption of anti-inflammatory foods and decreased intake of pro-inflammatory components—may represent an important component of comprehensive cardiovascular risk reduction strategies in T2D populations.

The energy adjustment incorporated within the E-DII construct enhances its methodological validity by accounting for between-person differences in total energy intake, thereby attenuating potential confounding biases arising from caloric intake variability [19]. In our cohort, the E-DII demonstrated superior predictive performance for mortality outcomes compared with the standard DII [specific model comparison statistics], suggesting that energy standardization meaningfully improves the epidemiological utility of dietary inflammatory indices in metabolic disease research.

As a simplified dietary assessment tool, the E-DII, like all dietary indices derived from self-reported dietary data, has important limitations. Specifically, the E-DII may not comprehensively capture the full complexity of individual dietary patterns and remains subject to measurement error and recall bias inherent in self-reported dietary assessment. Additionally, the 24-hour dietary recall may not fully represent usual dietary patterns, particularly in populations with highly variable day-to-day intake. These limitations may introduce non-differential misclassification and potentially attenuate observed associations. Future investigations should consider repeated dietary assessments over multiple seasons and employ more detailed dietary documentation methodologies to enhance the precision of dietary inflammatory quantification.

Furthermore, it is essential to distinguish the E-DII from socioeconomic status (SES), such as the Townsend Deprivation Index, although both serve as composite indices. While SES is a macro-level, upstream social determinant that encompasses income and education, the E-DII represents a micro-level, downstream behavioral exposure. Notably, diet often serves as a key mediatory pathway through which SES influences health outcomes. Unlike SES, which is systemic and difficult to modify at the individual level, dietary patterns are highly modifiable behaviors. By focusing on the E-DII, our study identifies a specific, actionable intervention target. This suggests that even for individuals in socioeconomically disadvantaged positions, improving dietary quality to reduce inflammatory potential could meaningfully mitigate adverse outcomes—a finding with significant implications for personalized clinical counseling and public health policy.

These findings carry important clinical implications. They emphasize that dietary counseling strategies for T2D patients should transcend conventional macronutrient-focused approaches to comprehensively address the inflammatory burden of the overall diet. The E-DII could be operationalized as a practical tool for dietary risk stratification, assisting clinicians in identifying high-risk individuals and facilitating goal-setting for dietary modification toward anti-inflammatory eating patterns such as the Mediterranean diet model. For individuals with prediabetes, while our findings did not demonstrate a significant independent association between E-DII and outcomes, the promotion of anti-inflammatory dietary patterns remains evidence-based and appropriate as part of comprehensive lifestyle modification aimed at T2D prevention and cardiometabolic health optimization.

Future investigations should prioritize replication of these findings in epidemiologically and ethnically diverse populations to establish the generalizability of our observations. Rigorously designed randomized controlled trials are needed to establish whether dietary interventions specifically targeting reduction of DII scores can effectively attenuate cardiovascular disease incidence and all-cause mortality risk in T2D populations. Additionally, mechanistic studies investigating the interrelationships among genetic inflammatory susceptibility, bacterial microecology, intestinal permeability, and dietary inflammatory load could elucidate the biological pathways through which dietary patterns influence cardiovascular outcomes in diabetes.

### Limitations

Despite its strengths, several limitations inherent to registry-based research must be acknowledged. First, as an observational study using UK Biobank data, we cannot entirely exclude residual confounding from unmeasured factors—such as genetic-environmental interactions or chronic stress—nor can we definitively establish causality. Second, dietary data were derived from self-reported 24-hour recalls, which are subject to recall bias and may not capture long-term dietary shifts throughout the 12-year follow-up. Third, the UK Biobank is characterized by a ‘healthy volunteer’ effect, with participants being generally more educated and healthier than the general population, which may limit the generalizability of our findings. Additionally, while outcomes were identified through linked electronic health records and death registries, the potential for coding errors or incomplete reporting remains. Finally, our results demonstrate associations rather than strict causation. These limitations underscore the need for future randomized controlled trials to confirm whether targeted anti-inflammatory dietary interventions can directly improve cardiovascular outcomes in prediabetic and diabetic populations.

## Conclusions

In summary, in patients with type 2 diabetes mellitus, significant correlations were observed between a pro-inflammatory diet and increased risks of cardiovascular events, cardiovascular mortality, and all-cause mortality. Therefore, optimizing the quality of the diet, especially in terms of reducing the inflammatory components, may be an effective means to reduce the risk of cardiovascular events, cardiovascular mortality, and all-cause mortality in patients with type 2 diabetes mellitus. To establish a causal relationship, more research is needed for verification. Such studies will not only enhance our understanding of the relationship between diet and chronic diseases, but they may also guide future clinical practice and provide more precise nutritional intervention strategies for patients with type 2 diabetes mellitus.

## Supporting information

S1 Table Subgroup analyses of the associations between the energy-adjusted DII and the risk of cardiovascular disease, cardiovascular mortality, and all-cause mortality in patients with prediabetes. (PDF)

S2 Table Subgroup analyses of the associations between the energy-adjusted DII and the risk of cardiovascular disease, cardiovascular mortality, and all-cause mortality in patients with type 2 diabetes mellitus. (PDF)
