# Supplementary material for: Associations of dietary inflammatory index with incident cardiovascular outcomes and all-cause mortality in patients with prediabetes and type 2 diabetes mellitus: A prospective study of data from the UK Biobank
Source: PLoS One. 2026 Nov 11;21(11):e0360954. doi: 10.1371/journal.pone.0360954 (PMC13754139; doi:10.1371/journal.pone.0360954)
Supplement: Supplementary file 1 [file pone.0360954.s001.pdf]

S1 Table. Subgroup analyses of the associations between the energy-adjusted DII and the risk of cardiovascular disease, cardiovascular mortality, and all-cause mortality in patients with prediabetes

Subgroups	Cardiovascular events	Cardiovascular mortality	All-cause mortality
**Age**			
**< 60 years (N=8494)**			
Anti-inflammatory	1.15 (0.95-1.39)	1.32 (0.54-3.23)	1.05 (0.79-1.40)
Neutral	Ref	Ref	Ref
Pro-inflammatory	1.00 (0.78-1.29)	0.97 (0.31-3.01)	1.13 (0.79-1.62)
**≥ 60 years (N=9699)**			
Anti-inflammatory	1.01 (0.90-1.14)	0.87 (0.51-1.49)	0.99 (0.83-1.18)
Neutral	Ref	Ref	Ref
Pro-inflammatory	0.95 (0.78-1.16)	0.90 (0.40-2.03)	1.23 (0.95-1.59)
**Sex**			
**Male (N=7924)**			
Anti-inflammatory	1.07 (0.94-1.22)	0.69 (0.39-1.25)	0.94 (0.77-1.15)
Neutral	Ref	Ref	Ref
Pro-inflammatory	0.93 (0.77-1.13)	0.93 (0.45-1.92)	1.21 (0.94-1.56)
**Female (N=10269)**			
Anti-inflammatory	1.02 (0.86-1.20)	2.01 (0.83-4.83)	1.09 (0.87-1.35)
Neutral	Ref	Ref	Ref
Pro-inflammatory	1.08 (0.82-1.41)	0.93 (0.19-4.56)	1.17 (0.82-1.66)
**BMI**			
**< 30 kg/m^2 (N=12167)**			
Anti-inflammatory	1.05 (0.93-1.20)	1.31 (0.74-2.33)	1.03 (0.85-1.23)
Neutral	Ref	Ref	Ref
Pro-inflammatory	1.09 (0.89-1.33)	1.47 (0.66-3.30)	1.30 (1.01-1.69)

**≥ 30 kg/m² (N=5976)**			
Anti-inflammatory	1.06 (0.89-1.25)	0.52 (0.22-1.22)	0.97 (0.76-1.24)
Neutral	Ref	Ref	Ref
Pro-inflammatory	0.83 (0.65-1.07)	0.48 (0.14-1.60)	1.05 (0.75-1.48)

Multivariate Cox proportional-hazards regression Model 1 was adjusted for age (continuous), sex, ethnicity, and Townsend deprivation index. Model 2 was adjusted for the variables adjusted in Model 1 plus BMI (categorical), physical activity, smoking status, alcohol intake frequency, sleep chronotype, and baseline HbA1c. For participants with diabetes mellitus, the model was adjusted for the variables adjusted in Model 2 plus insulin treatment and diabetes duration at baseline. BMI, body mass index; DII, Dietary Inflammatory Index; HbA1c, glycated hemoglobin.
