# Supplementary material for: Associations of dietary inflammatory index with incident cardiovascular outcomes and all-cause mortality in patients with prediabetes and type 2 diabetes mellitus: A prospective study of data from the UK Biobank
Source: PLoS One. 2026 Nov 11;21(11):e0360954. doi: 10.1371/journal.pone.0360954 (PMC13754139; doi:10.1371/journal.pone.0360954)
Supplement: Supplementary file 2 [file pone.0360954.s002.pdf]

S2 Table. Subgroup analyses of the associations between the energy-adjusted DII and the risk of cardiovascular disease, cardiovascular mortality, and all-cause mortality in patients with type 2 diabetes mellitus

Subgroups	Cardiovascular events	Cardiovascular mortality	All-cause mortality
**Age**			
**< 60 years (N=3083)**			
Anti-inflammatory	1.04 (0.80-1.35)	0.30 (0.07-1.34)	1.15 (0.75-1.76)
Neutral	Ref	Ref	Ref
Pro-inflammatory	1.45 (1.05-1.99)	1.76 (0.58-5.31)	1.52 (0.91-2.55)
**≥ 60 years (N=3386)**			
Anti-inflammatory	0.99 (0.84-1.18)	0.92 (0.52-1.61)	1.06 (0.84-1.34)
Neutral	Ref	Ref	Ref
Pro-inflammatory	1.08 (0.81-1.44)	1.34 (0.55-3.26)	1.31 (0.90-1.90)
**Sex**			
**Male (N=3914)**			
Anti-inflammatory	0.99 (0.83-1.17)	0.79 (0.44-1.42)	1.13 (0.89-1.42)
Neutral	Ref	Ref	Ref
Pro-inflammatory	1.17 (0.92-1.50)	1.53 (0.73-3.24)	1.50 (1.08-2.08)
**Female (N=2555)**			
Anti-inflammatory	1.09 (0.83-1.43)	0.73 (0.25-2.15)	0.96 (0.63-1.47)
Neutral	Ref	Ref	Ref
Pro-inflammatory	1.42 (0.94-2.15)	0.94 (0.19-4.73)	0.78 (0.35-1.76)
**BMI**			
**< 30 kg/m^2 (N=3100)**			
Anti-inflammatory	1.09 (0.87-1.35)	0.87 (0.42-1.80)	1.13 (0.84-1.53)
Neutral	Ref	Ref	Ref
Pro-inflammatory	1.40 (0.97-2.03)	2.57 (1.00-6.58)	1.76 (1.10-2.82)
**≥ 30 kg/m^2 (N=3348)**			

Anti-inflammatory	0.96 (0.79-1.16)	0.69 (0.33-1.44)	1.01 (0.76-1.34)
Neutral	Ref	Ref	Ref
Pro-inflammatory	1.14 (0.88-1.50)	1.04 (0.39-2.78)	1.14 (0.77-1.69)
**Duration of diabetes**			
**< 5 years (N=4314)**			
Anti-inflammatory	0.95 (0.79-1.14)	0.91 (0.47-1.77)	1.15 (0.89-1.49)
Neutral	Ref	Ref	Ref
Pro-inflammatory	1.04 (0.78-1.38)	1.45 (0.58-3.64)	1.25 (0.84-1.87)
**≥ 5 years (N=2155)**			
Anti-inflammatory	1.14 (0.91-1.44)	0.61 (0.26-1.42)	0.97 (0.69-1.36)
Neutral	Ref	Ref	Ref
Pro-inflammatory	1.57 (1.13-2.16)	1.42 (0.50-4.03)	1.61 (1.02-2.55)

Multivariate Cox proportional-hazards regression Model 1 was adjusted for age (continuous), sex, ethnicity, and Townsend deprivation index. Model 2 was adjusted for the variables adjusted in Model 1 plus BMI (categorical), physical activity, smoking status, alcohol intake frequency, sleep chronotype, and baseline HbA1c. For participants with diabetes mellitus, the model was adjusted for the variables adjusted in Model 2 plus insulin treatment and diabetes duration at baseline. BMI, body mass index; DII, Dietary Inflammatory Index; HbAlc, glycated hemoglobin.
